# Luteal-phase support in assisted reproductive technology: An ongoing challenge

**DOI:** 10.18502/ijrm.v19i9.9708

**Published:** 2021-10-10

**Authors:** Saeideh Dashti, Maryam Eftekhar

**Affiliations:** Research and Clinical Center for Infertility, Yazd Reproductive Sciences Institute, Shahid Sadoughi University of Medical Sciences, Yazd, Iran.

**Keywords:** Luteal-phase support, IVF, HCG, Progesterone, GnRH agonist, Recombinant LH.

## Abstract

It has been shown that in controlled ovarian hyper stimulation cycles, defective luteal phase is common. There are many protocols for improving pregnancy outcomes in women undergoing fresh and frozen in vitro fertilization cycles. These approaches include progesterone supplements, human chorionic gonadotropin, estradiol, gonadotropin-releasing hormone agonist, and recombinant luteinizing hormone. The main challenge is luteal-phase support (LPS) in cycles with gonadotropin-releasing hormone agonist triggering. There is still controversy about the optimal component and time for starting LPS in assisted reproductive technology cycles. This review aims to summarize the various protocols suggested for LPS in in vitro fertilization cycles.

## 1. Introduction

Normal luteal function is the main component for pregnancy maintenance. In natural ovulatory cycles, the corpus luteum can produce adequate progesterone after ovulation until the placental function starts at seven wk gestation. Any problems that disturb progesterone secretion in the secretory phase can lead to a defective luteal phase. Luteal-phase deficiency is a condition where there is insufficient endogenous progesterone for embryo implantation, which can be associated with infertility and pregnancy loss (1, 2).

Controlled ovarian stimulation techniques often induce endocrine defects in the luteal phase and evidence shows that luteal-phase dysfunction can cause lower pregnancy rates in in vitro fertilization cycles (3).

Luteal-phase support (LPS) is a well-known intervention for almost all stimulated assisted reproductive technology (ART) cycles. Ovarian stimulation cycles using both gonadotropin-releasing hormone (GnRH) agonist or antagonist protocols have been associated with a defective luteal phase that can disturb embryo implantation (4).

Multiple follicular development results in supraphysiological levels of estradiol and progesterone that have negative feedback on luteinizing hormone (LH)

secretion from the pituitary gland (5, 6). Other factors include the disruption of granulosa cell function after oocyte pick up, prolonged suppression of the pituitary after administration of GnRH agonist or GnRH antagonist, and the negative feedback of exogenous human chorionic gonadotropin (HCG) on the secretion of LH from the pituitary. It has been suggested that LH secretion is inhibited by the administration of HCG via a short-loop feedback mechanism (7, 8).

On the other hand, in frozen-thawed cycles endometrial preparation is completely induced by exogenous estrogen and progesterone because of the absence of a corpus luteum (9). Very different regimens are suggested for supporting the luteal phase in fresh and frozen-thawed cycles. However, the ideal approach, the right dose, and the best time for commencing are controversial. This review was planned to present the different aspects of LPS.

## 2. The luteal phase support in HCG triggered fresh transfer cycle

### Progesterone 

Exogenous progesterone supplementation has commonly been used for supporting the luteal phase; however, the route of progesterone administration is still controversial (10).

Progesterone helps the uterus to be quiescent by stabilizing lysosomal membranes, inhibiting prostaglandin synthesis, and reducing intracellular calcium concentration. On the other hand, progesterone plays a role as an immunomodulator and can facilitate implantation via improvement of endometrial receptivity. Also, progesterone induces decidual transformation through increased endometrial vascularity (11).

Evidence demonstrated that a “luteal gap” in progesterone secretion in the second part of the luteal phase that leads to insufficient luteinizing of the endometrium; exogenous progesterone can therefore be the first choice in such cases (12).

Optimal coordination between endometrial receptivity and the embryo is a key factor for successful implantation and evidence shows that progesterone can induce this process effectively. Progesterone supplementation for LPS is accessible in synthetic forms (17 alpha-hydroxy derivatives) as well as in a natural formulation (micronized) and can be administered by intramuscular (IM), oral, intravaginal, subcutaneous (SC), and transdermal modes (13).

It is recommended not to use 19-nortestosterone derivatives, such as medroxyprogesterone acetate and norethisterone acetate, for supporting the luteal phase because of androgen and corticoid effects (12).

#### Intramuscular progesterone

An IM injection is a common form of progesterone that is oil-based and has been used for decades. The usual dose of progesterone is 50 mg; however, doses have ranged from 25 to 100 mg daily (14). IM injection provides permanent serum levels of progesterone (with a peak concentration around 8 hr) and is thus considered to be a particularly effective option in some countries (especially in North America). Another advantage of IM progesterone is rapid absorption that results in high plasma concentrations after 2 hr (15).

It was showed that after a daily IM injection of 25 mg progesterone, a plasma concentration was achieved that was equivalent to that of the luteal phase in a natural cycle. The main limiting factors for using IM progesterone include side effects such as redness, pain, welts, infections, sterile abscesses, inflammatory response, and even rarely pulmonary complications (eosinophilic pneumonia), and also that it is an inconvenient route due to the required daily visits and injections (16-18). Due to these limitations, there is still debate about this route.

#### Vaginal progesterone

Because of the disadvantages of IM progesterone, researchers have looked for an alternative route that is more convenient and efficient; one such route is vaginal preparation, which is popular in medical facilities (19). Vaginal progesterone products are currently administered through several routes including pessaries (Cyclogest), capsules (Utrogestan), or tablets (Lutigest), gel (Crinone), and Inserts (Endometrine) (20).

Devroey and coworkers showed that the maximum serum concentration of progesterone was obtained after 3-8 hr of utilizing vaginal suppositories or tablet supplements and that administration of daily 300-600 mg daily of vaginal components resulted in sufficient available plasma levels (21).

The main advantage of vaginal progesterone products is “first uterine pass” progesterone is transported directly from the vagina to the uterus, which leads to adequate uterine tissue levels of progesterone with lower circulating levels (good bioavailability) (22). Evidence suggests that vaginal progesterone is the preferred route in ART cycles and should be considered as the first step in the administration of progesterone (16, 21).

Histopathologic evidence has shown that using 300-600 mg of vaginal micronized progesterone daily can induce similar endometrial maturation as 100 mg IM progesterone daily (14).

Vaginal progesterone gel was first approved because of its positive effects on donation cycles (23).

The micronized vaginal progesterone (MVP) product is available as a bio adhesive gel (Crinone 8% 90 mg) and in a fine powder. Substantial evidence suggests that Crinone is efficiently absorbed in the vagina and can cause suitable decidual transformation in the endometrium for successful implantation (14). First uterine pass is responsible for the significant therapeutic effect of the vaginal gel on the endometrium which is obtained 4-5 hr after application (24). A meta-analysis showed that using 90-mg of vaginal progesterone bio adhesive gel daily or 200-mg of progesterone capsules daily had a similar effect on LPS as using a 50-mg of IM progesterone daily (19). Similarly, in another study was reported the same clinical pregnancy rate between 400-mg twice daily progesterone vaginal pessaries and 90-mg daily progesterone vaginal gel (25). It seems that more studies are needed to determine the suitable dose for vaginal gel.

The new vaginal product that was introduced recently is the vaginal ring (Milprosa), that releases progesterone continuously. In phase III clinical trials it was shown that using a progesterone vaginal ring once a wk had no significant impact on pregnancy outcomes or major complications compared with using vaginal gel (8% Crinone) once a day starting from the day after oocyte pick up. However, a threefold higher rate of vaginal discharge was reported in the vaginal ring group (26). Ginsburg et al. explained that women preferred weekly vaginal rings to vaginal gel because they were more convenient with no interference with daily and sexual activities, even though the gel was less difficult and stressful to apply (27). The most common complaints about vaginal progesterone are increased vaginal discharge and irritation that can be avoided by rectal use.

In spite of this fact, it seems vaginal progesterone products are considered as the first choice for LPS at many centers because these products have few side effects and are easier to use by women than IM preparations (14).

A systematic review reported a similar potency, and safety between different formulations of vaginal products including Utrogestan Vaginal and Crinone, Cyclogest, and Endometrin (20).

#### Subcutaneous progesterone

Another injectable preparation of progesterone is a water-soluble subcutaneous (SC) self-administration component (Prolutex) that has been introduced as a good alternative for women who do not want to use vaginal or IM progesterone (14, 16). SC progesterone is commonly used in a dose of 25-50 mg daily (28, 29). However, in a randomized controlled trial, it was found that 25 mg daily SC progesterone can induce suitable decidual changes in the endometrium (30).

Overall, this new SC product is more convenient with a considerable level of satisfaction among women (31).

#### Oral progesterone

Oral administration of micronized progesterone has low bioavailability because of first-pass metabolism in the liver. Only 10% of oral micronized progesterone is absorbed, which leads to a lower pregnancy rate compared to vaginal or IM progesterone. Also, oral micronized progesterone is associated with nausea, drowsiness, and flushing (11, 22, 32). Additionally, evidence suggests that oral micronized progesterone does not induce the suitable predecidual changes in endometrial glands and stroma that are necessary for implantation (12).

One oral progesterone product that is currently in use is Dydrogesterone, which has a specific chemical structure that induces better bioavailability with suitable progesterone activity (33).

A meta-analysis showed that oral Dydrogesterone leads to higher clinical pregnancy rates compared to MVP (34).

A Cochrane Review demonstrated no differences in live birth rates or ongoing pregnancy rates between synthetic and micronized progesterone. However, women in the synthetic progesterone group had a higher clinical pregnancy rate than those in the micronized progesterone group (35).

A randomized clinical trial that was conducted at 37 IVF centers in 10 countries could not confirm the inferiority of oral Dydrogesterone compared with MVP and suggested that oral Dydrogesterone is a well-tolerated route of progesterone with a similar safety profile (36). Moreover, a phase-III randomized controlled trial (lotus II) compared oral Dydrogesterone (30 mg daily) with 8% MVP gel (90 mg daily from the day of ovum pick up until the 12${}^{\mathrm{th}}$ wk of gestation). They concluded that oral Dydrogesterone had the same efficacy and safety as MVP gel and can be a good choice for LPS (37). Because Dydrogesterone is a more convenient route with good tolerability, it has been approved for use in LPS in several countries. However, it should be considered that Dydrogesterone is a synthetic molecule which may have some epigenetic effects such as relating to congenital heart disease (38). Therefore, further investigations are needed.

#### Transdermal progesterone

Transdermal progesterone is not a good choice for LPS. The first reason is the need for a massive dose of progesterone for achieving the equal physiological effect. Another problem is the existence of a 5a-reductase enzyme in the skin that deactivates the important part of absorbed progesterone, leading to low levels of progesterone in circulation (12).

Finally, further studies are needed to investigate the proper dose of progesterone products. The ESHRE guideline stipulates the following doses for the different natural progesterone products: 50 mg once daily for IM progesterone, 25 mg daily for SC progesterone, 100 mg for the vaginal insert twice or thrice daily, 90 mg daily for vaginal gel, 600 mg daily for MVP in-oil capsules, and 400 mg twice daily for the vaginal pessary (39).

### HCG

HCG prompts the corpus luteum to produce progesterone continuously via mimicking LH pulsatility; therefore, for a long time many researchers believed that HCG should be the primary choice for LPS. However, this approach can increase the risk of ovarian hyper stimulation syndrome (OHSS) (16, 35). In a randomized controlled trial, was demonstrated that the administration of HCG for LPS did not improve the ongoing pregnancy rate in the natural frozen embryo transfer (FET) cycle (40). Evidence shows that after using HCG for the trigger in stimulated cycles, a gap of LH-like activity is expected at the time of implantation that can lead to a lower pregnancy rate. Several doses of HCG have been suggested for repairing this defect even though there is no universally-agreed opinion about this. Andersen and coworkers suggested that receiving continued low-dose HCG (e.g., 500 IU) prior to the mid-luteal phase can be efficient and convenient (2). The Cochrane Database of systematic reviews showed that administration of HCG or progesterone for LPS may improve live birth rates or ongoing pregnancy rates, although HCG, with or without progesterone, can increase the risk of OHSS (35).

### Estradiol

In natural ovulatory cycles, the corpus luteum secretes estrogen in addition to progesterone; therefore, some researchers have suggested that adding estrogen to progesterone for LPS could improve pregnancy outcomes. However, results from a meta-analysis did not support routine administration of estrogen along with progesterone for LPS in IVF cycles (11, 35). On the other hand, in antagonist cycles, serum estradiol may be diminished due to the increased level of serum progesterone, and this decrease is probably greater compared to serum estradiol in agonist cycles (41). Therefore, it has been suggested that adding estradiol in doses of 2-6 mg/day could help. However, a systematic review demonstrated that the addition of oral estradiol to progesterone supplementation in the luteal phase of IVF cycles did not improve outcomes (42). Similarly, two other studies revealed that utilizing different routes of estradiol including oral, transdermal patches, or transdermal gel for supporting the luteal phase in antagonist protocols did not improve pregnancy rates (42-44). There is still controversy surrounding the use of estradiol for LPS in fresh cycles and more studies should be conducted.

### Gonadotropin-releasing hormone agonist (GnRH-a)

The suggestion of using gonadotropin-releasing hormone agonist (GnRH-a) for LPS first emerged from inadvertent use of GnRH-a during the luteal phase. One study reported that accidental use of GnRH-a during the luteal phase improved implantation (45). Evidence explains that GnRH-a has an effect at three levels. Utilizing GnRH-a supports the corpus luteum through pituitary LH secretion. GnRH-a also has direct effects on the embryo and implantation process (11, 45, 46). Another possible mechanism is the effect that it has on trophectoderm cells and endometrial GnRH receptors (47, 48).

Local expression of endometrial receptors can activate endocrine-paracrine pathways leading to the secretion of angiogenic factors, growth factors, cytokines, and adhesion molecules. A meta-analysis showed that administration of a single-dose of GnRH-a (0.1 mg of triptorelin six days after intracytoplasmic sperm injection) increased the implantation rate in GnRH antagonist and long GnRH-a cycles, clinical pregnancy rate per transfer, and ongoing pregnancy rate in cycles with GnRH antagonist protocols (49). After using GnRH-a, no embryonic or fetal malformations related to this treatment were reported (45). Results from a meta-analysis in 2020 showed that the administration of GnRH-a for luteal support not only significantly improved the live birth rate, clinical pregnancy rate, and ongoing pregnancy rate, but also revealed a tendency to decrease the abortion rate (50). Another study showed that a single-dose of GnRH-a (triptorelin acetate, 0.1 mg) given on the sixth day after the oocyte pick up had a similar efficacy as three doses of HCG (51).

## 3. The luteal phase in GnRH-a-triggered fresh transfer cycles

In a natural ovulatory cycle, LH supports the corpus luteum to secrete estradiol and progesterone after ovulation. In controlled ovarian stimulation cycles, after HCG triggering, HCG mimics LH activity to motivate corpus luteum steroidogenesis. After the induction of final oocyte maturation with a bolus dose of GnRH-a, the duration of the luteal phase becomes shorter than the HCG cycle (9 days compared to 13 days) and the serum levels of progesterone and estradiol decrease in the luteal phase compared to the HCG cycle (52). As a consequence, significantly lower implantation rate are expected (53) and an intensive luteal phase is necessary to overcome this luteal phase insufficiency (54). Many studies have tried to find a more suitable replacement for LH with as few side effects as possible. One strategy is known as “the European approach” which involves utilizing HCG for rescuing the corpus luteum for the production of steroids (55).

### Dual trigger with low dose HCG and GnRH-a

Administration of low-dose HCG at the time of trigger rescues the corpus luteum for sufficient luteinization. In two studies was shown that adding 1,000 or 2,500 IU HCG (mean dose: 26.2 IU/kg) improved the pregnancy rate. They also reported a low OHSS rate in these patients (56, 57).

In order to avoid the risk of OHSS, it is reasonable to apply a low dose of HCG (1,000 IU) coupled with a GnRH-a trigger in women with a peak estradiol of < 4000 pg/ml. This regimen can improve the chance of implantation and live birth rates (54).

### Low-dose HCG at the time of ovum pick-up

Lower LH activity in the early luteal phase is known to be responsible for unacceptable results after the GnRH-a trigger. Maslow et al*.* showed that low-dose HCG can be used not only at the time of GnRH-a trigger but also 35 hr later, and both routes improve pregnancy rates. Women that received 1,000 IU HCG at the time of the GnRH-a trigger had equal live birth rates compared to those who received 1,500 IU HCG at the time of oocyte pick up (58). In two studies was suggested that it is crucial to determine an upper cutoff limit for those with > 25 follicles with a diameter of ≥ 11 mm before utilizing this strategy (59, 60).

### Low-dose HCG in the luteal phase

Two proof-of-concept studies reported that administration of 1,500 IU of HCG two or three days after ovum pick up can lead to higher mid-luteal progesterone levels (61, 62).

### Daily low dose recombinant HCG in the luteal phase

Another strategy suggested for supporting corpora lutea is using recombinant HCG (125 IU) beginning on either day two or six of stimulation and followed daily throughout the luteal phase (progesterone-free luteal phase). However, more studies are needed on this strategy (63).

### Luteal coasting

This strategy is based on findings that not all women after the GnRH-a trigger have to use aggressive LPS (64). In this method, the luteal phase steroid supplementation is not applied at first. When serum progesterone levels decrease, a bolus of 1,500 IU HCG is administered (63).

### Recombinant LH

The use of recombinant LH (rLH) for rescuing corpus luteum after the administration of GnRH-a for oocyte maturation was first introduced in a randomized study. Six repeated doses of 300 IU rLH was injected from the day of ovum pick up in addition to vaginal micronized progesterone. The main superiority of rLH to HCG is the shorter half-life of LH compared to HCG, which can minimize the risk of OHSS. Acceptable implantation rates were achieved with this novel rLH luteal supplementation scheme compared to the standard luteal progesterone protocol (65).

### Intensive luteal support (steroid-only luteal phase supplementation)

“The American approach” is the second policy that was introduced for LPS after the GnRH-a trigger. This strategy is based on using IM progesterone (50 mg daily) and transdermal estradiol (three 0.1 mg patches replaced every other day) for LPS (55, 66). In this method, corpus luteum function is ignored and the luteal phase is only supported by exogenous steroids. The main challenge in this route is the need for frequent monitoring of proestrone and estradiol to maintain the progesterone levels > 20 ng/mL and estradiol levels > 200 pg/mL along with LP (67, 68).

## 4. LPS for FET cycles

In FET cycles, neither estrogen nor progesterone is secreted from the corpus luteum; therefore, exogenous estradiol is necessary for proliferating the endometrium, which is followed by the administration of progesterone for supporting secretory endometrium. Synchronization between the embryo and endometrium is a key factor for successful implantation in FET cycles. The “window of implantation” is the ideal opportunity for trophoblast-endometrial cross-talking; it occurs around days 22-24 of a 28-day cycle (69).

Protocols used for frozen cycles consist of a natural cycle (NC), a modified natural cycle, and an artificial cycle (AC). In NC-FET, no steroid hormone is administered (70) and the best time for embryo transfer is determined by the observation of the LH surge; however, in modified NC-FET, embryo transfer is carried out after the induction of ovulation. Natural cycles can be a favored option in women with normal ovulatory menstrual cycles, although using luteal support in natural cycles is still controversial. Accordingly, it was revealed that an injection of 50 mg of IM progesterone twice daily starting from 36 hr after the HCG trigger was not effective in natural frozen-thawed cycles (71).

The most common route for the FET cycle is endometrial preparation by the administration of estradiol for achieving an endometrial thickness of approximately 0.8 cm and then adding progesterone for induction of secretory endometrium (69). An evaluation of the clinical factors affecting pregnancy outcomes in FET cycles showed that the duration of endometrial preparation did not influence pregnancy rates (72).

Usually, progesterone is utilized three to four days before a cleavage-stage embryo transfer and around five to six days before a blastocyst transfer. Casper and coworkers could not find any difference between IM and vaginal progesterone outcomes in this context (73). It seems pregnancy and live birth rates are comparable among all the variations in methods.

Estradiol can use as oral and, vaginal tablets, transdermal patches, SC or IM that studies did not find significant differences between them (74). Similarly, endometrial preparation can be induced by mild stimulation with exogenous gonadotropins or oral agents, but some researchers believe that ovarian stimulation may disturb endometrial vascularization and receptivity (75, 76). In a systematic review, was explained that clinical pregnancy rate and live birth rate in NC are comparable to mild ovarian stimulation using gonadotropins (76).

Then again, a meta-analysis conducted in 2013, showed that the usage of estrogen and progesterone did not suppress the pituitary completely and spontaneous luteinization sometimes occurred earlier. They suggested that the administration of GnRH-a may prevent follicular growth (AC-FET with GnRH-a) and spontaneous luteinization (77). Nevertheless an RCT in 2020 showed that the administration of GnRH-a prior to steroid preparation of the endometrium did not improve pregnancy rates in FET cycles in repeated implantation-failure group (78).

In spite of this, in an interventional pilot study, was demonstrated that using a single SC dose of 0.1 mg triptorelin at the time of implantation could have an immunological modulatory role in the endometrium. They suggested that administration of GnRH-a facilitates interaction between the endometrium and trophoblast and supports embryo implantation (79). However, the administration of 0.1 mg triptorelin acetate three days after embryo transfer (single dose) did not improve pregnancy outcomes (80).

A retrospective study has shown that using three dose of HCG in LPS can advance chemical and clinical pregnancy rate in the FET cycles (81). HCG is known as the first molecular message between the blastocyst and the decidua. Intrauterine HCG before embryo transfer may have beneficial effects on the endometrium in frozen-thawed cycles 82 was added (82).

## 5. The ideal time for initiating LPS

Knowing the appropriate time for starting LPS is currently challenging. In IVF cycles after HCG triggering, HCG levels decrease approximately five days after ovum pick up, which causes decreased levels of progesterone (83). Consequently, it is recommended that LPS is started before the endogenous progesterone diminishes; however, it is very important not to start early, as doing so can have a poor effect on endometrial receptivity. In a systematic review, Connell et al. showed that the optimal time for starting LPS is between 24 and 72 hr after ovum pick up (84).

However, a systematic review suggested that the day after oocyte pick up is the best time for initiation of LPS and that continuing more than three wk does not improve clinical pregnancy rates (85). The ESHRE guidelines recommended that LPS should be started in the interval time between the evening of the day of ovum pick up and day 3 post oocyte retrieval (39). Further research is needed to determine the best time for starting LPS.

## 6. Duration of luteal support

Many studies have evaluated the duration of LPS. One meta-analysis showed that continuing progesterone for two wk after a positive pregnancy test did not change miscarriage or delivery rates. Moreover, they found it is unnecessary to continue LPS up to 10 wk of pregnancy (5).

A systematic review conducted in 2019 explained that breaking up progesterone products in fresh IVF cycles with an HCG trigger after a positive HCG test can have a limited effect on pregnancy outcomes (7). Consequently, available evidence recommends that continuing progesterone products after the first positive pregnancy test is not necessary (14). The ESHRE guidelines recommend that LPS should be administered at least until the day of the pregnancy test (39).

## 7. Conclusion

It is well-known that LPS is a vital component in IVF cycles and several protocols have been suggested. However, there is still a debate about the best supplement, time for starting, route, and duration of LPS. The key factor is the personalization of treatment according to the patient's characteristics. This approach can help to optimize outcomes with the least side effects. Choosing a suitable LPS is very important in GnRH-a trigger cycles. More studies are needed to determine the optimal route and dosage for achieving successful outcomes.

##  Conflict of Interest

The authors declare that there is no conflict of interest.

## References

[B1] Practice Committee of the American Society for Reproductive Medicine

[B2] Andersen CY, Fischer R, Giorgione V, Kelsey ThW (2016). Micro-dose hCG as luteal phase support without exogenous progesterone administration: Mathematical modelling of the hCG concentration in circulation and initial clinical experience. J Assist Reprod Genet.

[B3] Casper RF, Yanushpolsky EH (2016). Optimal endometrial preparation for frozen embryo transfer cycles: Window of implantation and progesterone support. Fertil Steril.

[B4] Razieh DF, Maryam AR, Nasim T (2009). Beneficial effect of luteal-phase gonadotropin-releasing hormone agonist administration on implantation rate afterintracytoplasmic sperm injection. Taiwan J Obstet Gynecol.

[B5] Fatemi HM (2018). Simplifying luteal phase support in stimulated assisted reproduction cycles. Fertil Steril.

[B6] Leth-Moller K, Jagd SH, Humaidan P (2014). The luteal phase after GnRHa trigger-understanding an enigma. Int J Fertil Steril.

[B7] Watters M, Noble M, Child T, Nelson S (2019). Short versus extended progesterone supplementation for luteal phase support in fresh IVF cycles: A systematic review and meta-analysis. Reprod BioMed Online.

[B8] Miyake A, Aono T, Kinugasa T, Tanizawa O, Kurachi K (1979). Suppression of serum levels of luteinizing hormone by short-and long-loop negative feedback in ovariectomized women. J Endocrinol.

[B9] Griesinger G, Meldrum D (2018). Introduction: Management of the luteal phase in assisted reproductive technology. Fertil Steril.

[B10] Suthaporn S, Jayaprakasan K, Maalouf W, Thornton JG, Walker KF (2020). The strength of evidence supporting luteal phase progestogen after assisted reproduction: A systematic review with reference to trial registration and pre-specified endpoints. Eur J Obstet Gynecol Reprod Biol.

[B11] Nigam A (2018). Luteal phase support: Why, when and how. Pan Asian J Obs Gyn.

[B12] de Ziegler D, Pirtea P, Andersen CY, Ayoubi JM (2018). Role of gonadotropin-releasing hormone agonists, human chorionic gonadotropin (hCG), progesterone, and estrogen in luteal phase support after hCG triggering, and when in pregnancy hormonal support can be stopped. Fertil Steril.

[B13] Zarei A, Sohail P, Parsanezhad ME, Alborzi S, Samsami A, Azizi M (2017). Comparison of four protocols for luteal phase support in frozen-thawed Embryo transfer cycles: A randomized clinical trial. Arch Gynecol Obstet.

[B14] Yanushpolsky EH (2015). Luteal phase support in in vitro fertilization. Semin Reprod Med.

[B15] Nillius SJ, Johansson ED (1971). Plasma levels of progesterone after vaginal, rectal, or intramuscular administration of progesterone. Am J Obstet Gynecol.

[B16] Lawrenz B, Coughlan C, Fatemi HM (2019). Individualized luteal phase support. Curr Opin Obstet Gynecol.

[B17] Bernardo-Escudero R, CortÃ©s-Bonilla M, Alonso-Campero R, de JesÃºs Francisco-Doce MT, ChavarÃ­n-GonzÃ¡lez J, Pimentel-MartÃ­nez S, et al (2012). Observational study of the local tolerability of injectable progesterone microspheres. Gynecol Obstet Invest.

[B18] Veysman B, Vlahos I, Oshva L (2006). Pneumonitis and eosinophilia after in vitro fertilization treatment. Ann Emerg Med.

[B19] Zarutskie PW, Phillips JA (2009). A meta-analysis of the route of administration of luteal phase support in assisted reproductive technology: Vaginal versus intramuscular progesterone. Fertil Steril.

[B20] Child T, Leonard SA, Evans JS, Lass A (2018). Systematic review of the clinical efficacy of vaginal progesterone for luteal phase support in assisted reproductive technology cycles. Reprod Biomed Online.

[B21] Devroey P, Palermo G, Bourgain C, Van Waesberghe L, Smitz J, Van Steirteghem A (1989). Progesterone administration in patients with absent ovaries. Int J Fertil.

[B22] Tavaniotou A, Smitz J, Bourgain C, Devroey P (2000). Comparison between different routes of progesterone administration as luteal phase support in infertility treatments. Hum Reprod Update.

[B23] Gibbons WE, Toner JP, Hamacher P, Kolm P (1998). Experience with a novel vaginal progesterone preparation in a donor oocyte program. Fertil Steril.

[B24] Chi H, Li R, Qiao J, Chen X, Wang X, Hao G, et al (2019). Vaginal progesterone gel is non-inferior to intramuscular progesterone in efficacy with acceptable tolerability for luteal phase support: A prospective, randomized, multicenter study in China. Eur J Obstet Gynecol Reprod Biol.

[B25] Saunders H, Khan C, D’Hooghe Th, MagnÃºsdÃ³ttir ThB, Klingmann I, HrafnsdÃ³ttir S, et al (2020). Efficacy, safety and tolerability of progesterone vaginal pessaries versus progesterone vaginal gel for luteal phase support after in vitro fertilisation: A randomised controlled trial. Hum Reprod.

[B26] Stadtmauer L, Silverberg KM, Ginsburg ES, Weiss H, Howard B (2013). Progesterone vaginal ring versus vaginal gel for luteal support with in vitro fertilization: A randomized comparative study. Fertil Steril.

[B27] Ginsburg ES, Jellerette-Nolan T, Daftary G, Du Y, Silverberg KM (2018). Patient experience in a randomized trial of a weekly progesterone vaginal ring versus a daily progesterone gel for luteal support after in vitro fertilization. Fertil Steril.

[B28] Doblinger J, Cometti B, Trevisan S, Griesinger G (2016). Subcutaneous progesterone is effective and safe for luteal phase support in IVF: An individual patient data meta-analysis of the phase III trials. PloS One.

[B29] de Ziegler D, Sator M, Binelli D, Leuratti Ch, Cometti B, Bourgain C, et al (2013). A randomized trial comparing the endometrial effects of daily subcutaneous administration of 25 mg and 50 mg progesterone in aqueous preparation. Fertil Steril.

[B30] Lockwood G, Griesinger G, Cometti B (2014). Subcutaneous progesterone versus vaginal progesterone gel for luteal phase support in in vitro fertilization: A noninferiority randomized controlled study. Fertil Steril.

[B31] Venturella R, Vaiarelli A, Buffo L, D’alessandro P, Colamaria S, Pedri S, et al (2018). Progesterone for preparation of the endometrium for frozen-thawed blastocyst transfer in vitro fertilization cycles: A prospective study on patients’ opinions on a new subcutaneous formulation. Gynecol Endocrinol.

[B32] Licciardi FL, Kwiatkowski A, Noyes NL, Berkeley AS, Krey LL, Grifo JA (1999). Oral versus intramuscular progesterone for in vitro fertilization: A prospective randomized study. Fertil Steril.

[B33] Schindler AE

[B34] Barbosa MWP, Silva LR, Navarro PA, Ferriani RA, Nastri CO, Martins WP (2016). Dydrogesterone vs progesterone for luteal-phase support: Systematic review and meta-analysis of randomized controlled trials. Ultrasound Obstet Gynecol.

[B35] van der Linden M, Buckingham K, Farquhar C, Kremer JA, Metwally M (2015). Luteal phase support for assisted reproduction cycles. Cochrane Database Syst Rev.

[B36] Griesinger G, Blockeel Ch, Sukhikh GT, Patki A, Dhorepatil B, Yang DZ, et al (2018). Oral dydrogesterone versus intravaginal micronized progesterone gel for luteal phase support in IVF: A randomized clinical trial. Hum Reprod.

[B37] Yang DZ, Griesinger G, Wang W, Gong F, Liang X, Zhang H, et al (2020). A Phase III randomized controlled trial of oral dydrogesterone versus intravaginal progesterone gel for luteal phase support in in vitro fertilization (Lotus II): Results from the Chinese mainland subpopulation. Gynecol Endocrinol.

[B38] Zaqout M, Aslem E, Abuqamar M, Abughazza O, Panzer J, De Wolf D (2015). The impact of oral intake of dydrogesterone on fetal heart development during early pregnancy. Pediatr Cardiol.

[B39] The Eshre Guideline Group On Ovarian Stimulation, Bosch E, Broer S, Griesinger G, Grynberg M, Humaidan P, et al (2020). ESHRE guideline: Ovarian stimulation for IVF/ICSI. Hum Reprod Open.

[B40] Lee VCY, Li RHW, Yeung WSB, Pak Chung HO, Ng EHY (2017). A randomized double-blinded controlled trial of hCG as luteal phase support in natural cycle frozen embryo transfer. Hum Reprod.

[B41] Tavaniotou A, Devroey P (2006). Luteal hormonal profile of oocyte donors stimulated with a GnRH antagonist compared with natural cycles. Reprod Biomed Online.

[B42] Pinheiro LMA, da Silva CÃ¢ndido P, Moreto TC, Di Almeida WG, de Castro EC (2017). Estradiol use in the luteal phase and its effects on pregnancy rates in IVF cycles with GnRH antagonist: A systematic review. JBRA Assist Reprod.

[B43] Scheffer JB, Scheffer BB, de Carvalho RF, Aguiar AP, Lozano DHM, Labrosse J, et al (2019). A comparison of the effects of three luteal phase support protocols with estrogen on in vitro fertilization-embryo transfer outcomes in patients on a GnRH antagonist protocol. JBRA Assist Reprod.

[B44] Munjal R, Gupta S (2019). Addition of oestradiol to progesterone for luteal phase support in GnRh antagonist IVF/ICSI cycles. Fertil Sci Res.

[B45] Tesarik J, Mendoza-Tesarik R, Mendoza N (2016). Gonadotropin-releasing hormone agonist for luteal phase support: The origin of the concept, current experience, mechanism of action and future perspectives. Fertil Steril.

[B46] Tesarik J, Hazout A, Mendoza C (2004). Enhancement of embryo developmental potential by a single administration of GnRH agonist at the time of implantation. Hum Reprod.

[B47] ÅžÃ¼kÃ¼r YE, ÅžimÅŸir C, Ã–zdemir ED, SÃ¶nmezer M (2016). GnRH agonist addition to routine luteal phase support in assisted reproductive technology. Austin J In Vitro Fertil.

[B48] Pirard C, Loumaye E, Laurent P, Wyns Ch (2015). Contribution to more patient-friendly ART treatment: Efficacy of continuous low-dose GnRH agonist as the only luteal support-results of a prospective, randomized, comparative study. Int J Endocrinol.

[B49] Oliveira JBA, Baruffi R, Petersen CG, Mauri AL, Cavagna M, Franco Jr JG (2010). Administration of single-dose GnRH agonist in the luteal phase in ICSI cycles: A meta-analysis. Reprod Biol Endocrinol.

[B50] Ma X, Du W, Hu J, Yang Y, Zhang X (2020). Effect of gonadotrophin-releasing hormone agonist addition for luteal support on pregnancy outcome in vitro fertilization/intracytoplasmic sperm injection cycles: A meta-analysis based on randomized controlled trials. Gynecol Obstet Invest.

[B51] Yilmaz NK, Kara M, HanÃ§erlioÄŸullari N, ErkilinÃ§ S, CoÅŸkun B, Sargin A, et al (2018). Analysis of two different luteal phase support regimes and evaluation of in vitro fertilization-intra cytoplasmic sperm injection outcomes. Turk J Obstet Gynecol.

[B52] Beckers NGM, Macklon NS, Eijkemans MJ, Ludwig M, Felberbaum RE, Diedrich K, et al (2003). Nonsupplemented luteal phase characteristics after the administration of recombinant human chorionic gonadotropin, recombinant luteinizing hormone, or gonadotropin-releasing hormone (GnRH) agonist to induce final oocyte maturation in in vitro fertilization patients after ovarian stimulation with recombinant follicle-stimulating hormone and GnRH antagonist cotreatment. J Clin Endocrinol Metab.

[B53] Engmann L, Benadiva C (2005). GnRH agonist (buserelin) or hCG for ovulation induction in GnRH antagonist IVF/ICSI cycles: A prospective randomized study. Hum Reprod.

[B54] Benadiva C, Engmann L

[B55] Dosouto C, Haahr Th, Humaidan P (2017). Gonadotropin-releasing hormone agonist (GnRHa) trigger-state of the art. Reprod Biol.

[B56] Shapiro BS, Daneshmand ST, Garner FC, Aguirre M, Thomas Sh (2008). Gonadotropin-releasing hormone agonist combined with a reduced dose of human chorionic gonadotropin for final oocyte maturation in fresh autologous cycles of in vitro fertilization. Fertil Steril.

[B57] Shapiro BS, Daneshmand ST, Garner FC, Aguirre M, Hudson C (2011). Comparison of “triggers” using leuprolide acetate alone or in combination with low-dose human chorionic gonadotropin. Fertil Steril.

[B58] Maslow BL, Griffin D, Benadiva CA, Nulsen J, Engmann L

[B59] Castillo JC, Haahr Th, MartÃ­nez-Moya M, Humaidan P (2020). Gonadotropin-releasing hormone agonist for ovulation trigger-OHSS prevention and use of modified luteal phase support for fresh embryo transfer. Ups J Med Sci.

[B60] Ata B, Seyhan A, Polat M, Son WY, Yarali H, Dahan M (2013). GnRHa trigger and modified luteal support with one bolus of hCG should be used with caution in extreme responder patients. Hum Reprod.

[B61] Vanetik Sh, Segal L, Breizman T, Kol Sh (2018). Day two post retrieval 1500 IUI hCG bolus, progesterone-free luteal support post GnRH agonist trigger-a proof of concept study. Gynecol Endocrinol.

[B62] Haas J, Kedem A, Machtinger R, Dar Sh, Hourvitz A, Yerushalmi G, et al (2014). HCG (1500IU) administration on day 3 after oocytes retrieval, following GnRH-agonist trigger for final follicular maturation, results in high sufficient mid luteal progesterone levels-a proof of concept. J Ovarian Res.

[B63] Andersen CY, Elbaek HO, Alsbjerg B, Laursen RJ, Povlsen BB, Thomsen L, et al (2015). Daily low-dose hCG stimulation during the luteal phase combined with GnRHa triggered IVF cycles without exogenous progesterone: A proof of concept trial. Hum Reprod.

[B64] Kol Sh, Breyzman T, Segal L, Humaidan P (2015). ‘Luteal coasting’ after GnRH agonist trigger-individualized, HCG-based, progesterone-free luteal support in ‘high responders’: A case series. Reprod Biomed Online.

[B65] Papanikolaou EG, Verpoest W, Fatemi H, Tarlatzis B, Devroey P, Tournaye H (2011). A novel method of luteal supplementation with recombinant luteinizing hormone when a gonadotropin-releasing hormone agonist is used instead of human chorionic gonadotropin for ovulation triggering: A randomized prospective proof of concept study. Fertil Steril.

[B66] Humaidan P, Engmann L, Benadiva C (2015). Luteal phase supplementation after gonadotropin-releasing hormone agonist trigger in fresh embryo transfer: The American versus European approaches. Fertil Steril.

[B67] Iliodromiti S, Lan VTN, Tuong HM, Tuan PhH, Humaidan P, Nelson SM (2013). Impact of GnRH agonist triggering and intensive luteal steroid support on live-birth rates and ovarian hyperstimulation syndrome: A retrospective cohort study. J Ovarian Res.

[B68] Engmann L, DiLuigi A, Schmidt D, Nulsen J, Maier D, Benadiva C (2008). The use of gonadotropin-releasing hormone (GnRH) agonist to induce oocyte maturation after cotreatment with GnRH antagonist in high-risk patients undergoing in vitro fertilization prevents the risk of ovarian hyperstimulation syndrome: A prospective randomized controlled study. Fertil Steril.

[B69] Groenewoud ER (2017). Endometrial preparation methods in frozen-thawed embryo transfer.

[B70] Shiba R, Kinutani M, Okano Sh, Kawano R, Kikkawa Y (2020). Efficacy of four vaginal progesterones for luteal phase support in frozen-thawed embryo transfer cycles: A randomized clinical trial. Reprod Med Biol.

[B71] Eftekhar M, Rahsepar M, Rahmani E (2013). Effect of progesterone supplementation on natural frozen-thawed embryo transfer cycles: A randomized controlled trial. Int J Fertil Steril.

[B72] Eftekhar M, Rahmani E, Pourmasumi S (2014). Evaluation of clinical factors influencing pregnancy rate in frozen embryo transfer. Iran J Reprod Med.

[B73] Casper RF

[B74] Corroenne R, El Hachem H, Verhaeghe C, Legendre G, Dreux C, Jeanneteau P, et al (2020). Endometrial preparation for frozen-thawed embryo transfer in an artificial cycle: Transdermal versus vaginal estrogen. Sci Rep.

[B75] Fatemi HM, Popovic-Todorovic B (2013). Implantation in assisted reproduction: A look at endometrial receptivity. Reprod Biomed Online.

[B76] Yarali H, Polat M, Mumusoglu S, Yarali I, Bozdag G (2016). Preparation of endometrium for frozen embryo replacement cycles: A systematic review and meta-analysis. J Assist Reprod Genet.

[B77] Groenewoud ER, Cantineau AEP, Kollen BJ, Macklon NS, Cohlen BJ (2013). What is the optimal means of preparing the endometrium in frozen-thawed embryo transfer cycles? A systematic review and meta-analysis. Hum Reprod Update.

[B78] Davar R, Dashti S, Omidi M

[B79] Seikkula J, Ahinko K, Polo-Kantola P, Anttila L, Hurme S, Tinkanen H, et al (2018). Mid-luteal phase gonadotropin-releasing hormone agonist support in frozen-thawed embryo transfers during artificial cycles: A prospective interventional pilot study. J Gynecol Obstet Hum Reprod.

[B80] Ye H, Luo X, Pei L, Li F, Li Ch, Chen Y, et al (2019). The addition of single dose GnRH agonist to luteal phase support in artificial cycle frozen embryo transfer: A randomized clinical trial. Gynecol Endocrinol.

[B81] Eftekhar M, Dashti S, Omidi M, Tabatabaei AA

[B82] Huang P, Wei L, Li X (2017). A study of intrauterine infusion of human chorionic gonadotropin (hCG) before frozen-thawed embryo transfer after two or more implantation failures. Gynecol Endocrinol.

[B83] Beckers NGM, Laven JSE, Eijkemans MJC, Fauser BCJM (2000). Follicular and luteal phase characteristics following early cessation of gonadotrophin-releasing hormone agonist during ovarian stimulation for in-vitro fertilization. Hum Reprod.

[B84] Connell MT, Szatkowski JM, Terry N, DeCherney AH, Propst AM, Hill MJ (2015). Timing luteal support in assisted reproductive technology: A systematic review. Fertil Steril.

[B85] Mohammed A, Woad KJ, Mann GE, Craigon J, Raine-Fenning N, Robinson RS (2019). Evaluation of progestogen supplementation for luteal phase support in fresh in vitro fertilization cycles. Fertil Steril.

